# A Timing-Based Split-Path Sensing Circuit for STT-MRAM

**DOI:** 10.3390/mi13071004

**Published:** 2022-06-26

**Authors:** Bayartulga Ishdorj, Jeongyeon Kim, Jae Hwan Kim, Taehui Na

**Affiliations:** Department of Electronics Engineering, Incheon National University, Incheon 22012, Korea; tulga5111@gmail.com (B.I.); jyeon117@inu.ac.kr (J.K.); asrgzone@naver.com (J.H.K.)

**Keywords:** dynamic reference voltage, read disturbance, read yield, sense amplifier, sensing circuit, spin-transfer torque magnetoresistive random access memory (STT-MRAM)

## Abstract

Spin-transfer torque magnetoresistive random access memory (STT-MRAM) applications have received considerable attention as a possible alternative for universal memory applications because they offer a cost advantage comparable to that of a dynamic RAM with fast performance comparable to that of a static RAM, while solving the scaling issues faced by conventional MRAMs. However, owing to the decrease in supply voltage (*V*_DD_) and increase in process fluctuations, STT-MRAMs require an advanced sensing circuit (SC) to ensure a sufficient read yield in deep submicron technology. In this study, we propose a timing-based split-path SC (TSSC) that can achieve a greater read yield compared to a conventional split-path SC (SPSC) by employing a timing-based dynamic reference voltage technique to minimize the threshold voltage mismatch effects. Monte Carlo simulation results based on industry-compatible 28-nm model parameters reveal that the proposed TSSC method obtains a 42% higher read access pass yield at a nominal *V*_DD_ of 1.0 V compared to the SPSC in terms of iso-area and -power, trading off 1.75× sensing time.

## 1. Introduction

To prolong the battery life in lithium-ion battery-powered applications, such as smartphones, wearable devices, and wireless sensor nodes, it is crucial to achieve good performance with low power consumption [1]. Even though a conventional magnetoresistive random access memory (MRAM) has the speed of a static RAM (SRAM) and density of a dynamic RAM (DRAM), it has unique problems, including poor scalability and excessive power consumption owing to large write currents. Therefore, spin-transfer torque MRAM (STT-MRAM) has emerged as the top choice for universal memory applications owing to its short access time, low power consumption, and high density [2]. In addition, STT-MRAM has outstanding scalability, as the critical switching current of the magnetic tunnel junction (MTJ) decreases with device size to overcome the scaling problems faced by conventional memories, such as DRAM, SRAM, and flash memories [3,4,5]. In other words, STT-MRAM can achieve a higher performance than DRAM and smaller cell size than SRAM, with the nonvolatility of flash memory [6,7,8,9].

However, STT-MRAM faces a read yield degradation problem when used in deep submicron technologies because of the large process variations in low supply voltage (*V*_DD_) and small resistance difference between the low resistance (state 0) and high resistance (state 1) states of the MTJ [10,11]. The current (Δ*I*_0_ or Δ*I*_1_) or voltage differences (Δ*V*_0_ or Δ*V*_1_) generated in a sensing circuit (SC), which are then conveyed to a sense amplifier (SA), can be expressed as:

(1) $$\Delta I_{0,1}=\left|I_{\mathrm{data}0,1}-I_{\mathrm{ref}}\right|$$

(2) $$\Delta V_{0,1}=\left|V_{\mathrm{data}0,1}-V_{\mathrm{ref}}\right|=\Delta I_{0,1}\cdot r_{O\_\mathrm{PLD}}$$

where *I*_data0_ (*I*_data1_) and *I*_ref_ are the currents flowing through the data cell in state 0 (state 1) and reference cell, respectively. *V*_data0_ (*V*_data1_) and *V*_ref_ are the output voltages of the data and reference branches in the SC, respectively, and *r*_O_PLD_ is the output resistance of the load PMOS. Assuming a fixed *V*_ref_, Δ*V*_0,1_ is approximately half of the difference between *V*_data0_ and *V*_data1_ because *V*_ref_ is ideally equal to (*V*_data0_ + *V*_data1_)/2 [10]. To overcome the read yield degradation, a higher Δ*V*_0,1_ and lower impact of process variation are required in deep submicron technology nodes. However, Δ*V*_0,1_ is limited by *V*_DD_ and current, and the variations in the process parameters inevitably increase as the technology node scales down.

Furthermore, the offset voltage of the SA (*V*_SA_OS_) must be considered for a successful read operation. Thus, Δ*V*_0,1_ must be larger than *V*_SA_OS_ to generate the read-access path. The statistical distributions between Δ*V*_0,1_ and *V*_SA_OS_ can be modeled using a Gaussian distribution [11]. The read-access pass yield for a single cell with states 0 or 1 (*RAPY*_CELL0,1_) [12] is expressed as

(3) $$RAPY_{\mathrm{CELL}0,1}=\frac{\mu_{\Delta V0,1}-\mu_{\mathrm{SA}\_\mathrm{OS}}}{\sqrt{\sigma_{\Delta V0,1}^{2}-\sigma_{\mathrm{SA}\_\mathrm{OS}}^{2}}}$$

where *μ*_ΔV0,1_ and *μ*_SA_OS_ are the means of Δ*V*_0,1_ and *V*_SA_OS_, respectively, and *σ*_ΔV0,1_ and *σ*_SA_OS_ are the standard deviations of Δ*V*_0,1_ and *V*_SA_OS_, respectively. *RAPY*_CELL_ is defined as the minimum value between *RAPY*_CELL0_ and *RAPY*_CELL1_.

(4) $$RAPY_{\mathrm{CELL}}=\min(RAPY_{\mathrm{CELL}0},\;RAPY_{\mathrm{CELL}1})$$

In this study, a novel timing-based split-path sensing circuit (TSSC) that is tolerant to process variations and increases Δ*V*_0,1_ value is proposed and compared with various SCs with respect to *RAPY*_CELL_, delay, and power consumption. It improves *μ*_ΔV0,1_ using the dynamic reference voltage (DRV) technique that modifies *V*_ref_ according to the MTJ state. It also reduces *σ*_ΔV0,1_ by compensating the transistor mismatch and effectively increasing the sensing current. Even though the split-path sensing circuit (SPSC) [10] is considered to have optimal performance in terms of read yield, Monte Carlo HSPICE simulation results based on industry-compatible 28-nm model parameters reveal that the proposed TSSC achieves a 42% boost in *RAPY*_CELL_ at a *V*_DD_ of 1.0 V when compared to the SPSC in terms of iso-area and -power. The remainder of this paper is organized as follows: Section 2 describes the operational principles and characteristics of the conventional SCs and proposed TSSC. Section 3 compares the performance of the proposed SC and conventional SCs. Finally, Section 4 presents the conclusions drawn from our study.

## 2. Previous SCs and Proposed TSSC

In this section, the characteristics and operation principles of the existing and proposed SCs are described.

### 2.1. Existing SCs

**Figure 1:**
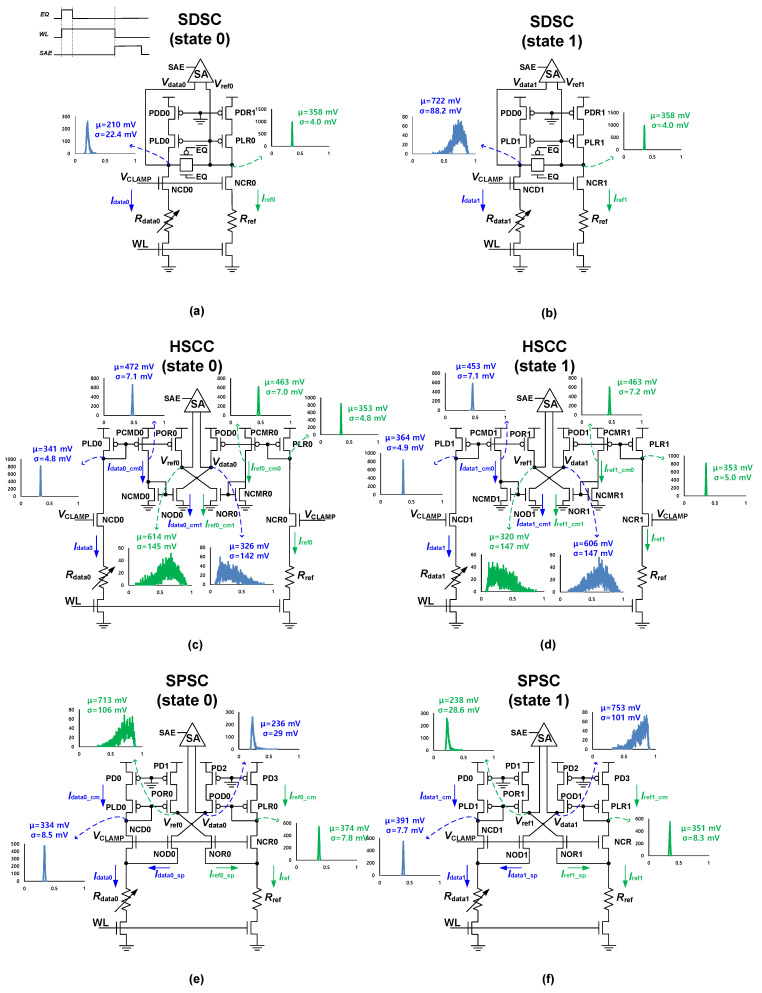
Schematics of conventional SCs with voltage distributions and timing diagrams. (**a**) SDSC [11] at state 0; (**b**) SDSC at state 1; (**c**) HSCC [13] at state 0; (**d**) HSCC at state 1; (**e**) SPSC [10] at state 0; (**f**) SPSC at state 1.

Three conventional SCs [10,11,13] for STT-MRAM are shown in Figure 1 along with their output voltage distributions. To analyze the voltage distributions, industry-compatible 28-nm model parameter libraries were used. Temperature was set to room temperature (25 °C) and, to compare in terms of iso-area and -power, the layout area of SCs were set to be identical by varying the transistor sizes. In addition, the gate voltage of the clamp NMOS (*V*_CLAMP_) was used differently for each SC to set the sensing current to 20 μA at state 1.

**Figure 2:**
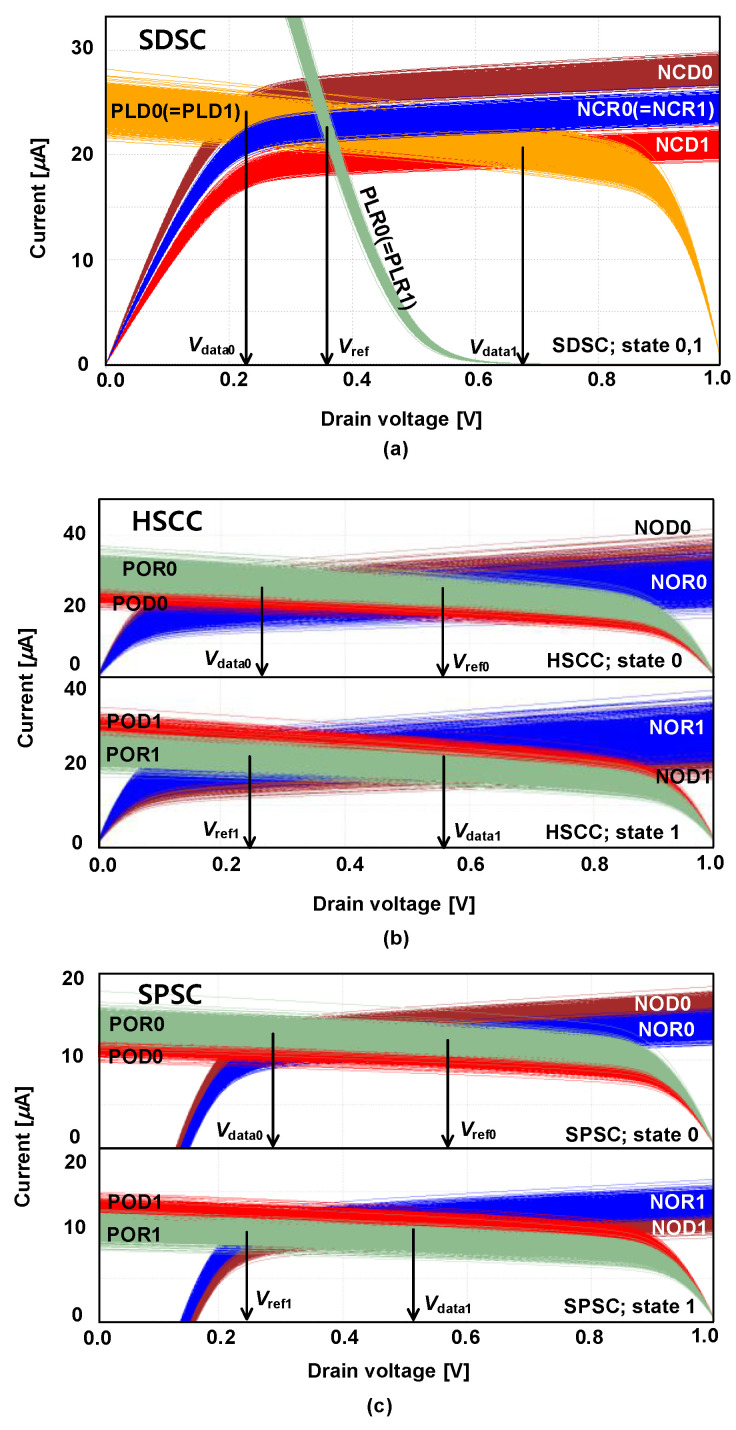
I-V curves of the conventional SCs with both MOSFET and MTJ process variations for analyzing operating point. (**a**) SDSC at states 0 and 1; (**b**) HSCC states 0 and 1; (**c**) SPSC at states 0 and 1.

Figure 1a,b illustrate the schematics and output voltage distributions of the source degeneration sensing circuit (SDSC) [11] at states 0 and 1, respectively. In the SDSC, the clamp NMOS (NCD, NCR) is used to generate Δ*I*, which is then converted to Δ*V* via a load PMOS (PLD, PLR) using a current mirror; then, Δ*V* is fed to the SA. In addition, SDSC achieves a PMOS (PDD, PDR) degeneration between *V*_DD_ and load PMOS for source degeneration purposes. The source degeneration effect reduces the variation in the load PMOS and increases *r*_O_PLD_, leading to an improvement in the *RAPY*_CELL_. The I-V curve shown in Figure 2a represents the relationship between the drain voltage of each MOSFET and current through each MOSFET in the SDSC. The crossing points of the I-V curve for the load PMOS and clamp NMOS denote the operating points. For example, the crossing points for PLR and NCR, PLD0 and NCD0, and PLD1 and NCD1 are the operating points for *V*_ref_, *V*_data0_, and V_data1_, respectively. PLD0 (PLD1) passes through the operating point between PLR and NCR, as *V*_ref_ denotes the gate voltage of PLD0 (PLD1). The drain voltage distribution of the PLR shown in Figure 1a has a small standard deviation because of the large slope (i.e., the output resistance is small) of the diode-connected PLR. When the output resistance is small, the voltage variation is small as well, expressed in (2). The PLD has a relatively large standard deviation owing to the small slope of the PLD I-V curve and current mirror variation. Thus, to obtain a proper sensing margin in SDSC, the PLD variation must be reduced. SDSC can obtain an adequate read yield in 65-nm process technology because the degeneration effect reduces the process variation in the SC. However, under 65-nm process technology, the SDSC suffers from significant read yield degradation because the fixed *V*_ref_ limits *μ*_ΔV0,1_ and process variation increment increases *σ*_ΔV0,1_.

Figure 1c,d illustrate the SC with a highly symmetric cross-coupled current mirror (HSCC) [13] at states 0 and 1, respectively. To address the fixed *V*_ref_ issue observed in SDSC, the HSCC uses the DRV technique that adjusts *V*_ref_ according to the data state to enhance the sensing margin [14]. Figure 2b shows the I-V curve of HSCC, where crossing point of POR and NOR transistors’ I-V curves represents *V*_ref_. As demonstrated, *V*_ref_ decreases at state 1 (*V*_ref1_) and increases at state 0 (*V*_ref0_). The DRV technique can almost double *μ*_ΔV0,1_ compared to the fixed *V*_ref_ approach used in the SDSC. However, in the HSCC, three current mirrors from the PLD to the NCMD are employed to generate *V*_data0,1_. Consequently, these current mirrors induce a substantial current mismatch, which increases *σ*_ΔV0,1_. Neglecting the channel-length modulation, the current through the NCD (*I*_NCD_) for the current mirror is expressed as

(5) $$I_{\mathrm{NCD}}=A{\left(V_{\mathrm{GS}}-V_{\mathrm{TH}}\right)}^{2}$$

where *A* is the transconductance parameter, *V*_GS_ is the gate-to-source voltage of the NCD, and *V*_TH_ is the threshold voltage. The current through the PCMD (*I*_data_cm0_), including the mismatch effects, is given by

(6) $$I_{\mathrm{data}\_\mathrm{cm}0}=(A+\Delta A){\left(V_{\mathrm{GS}}-V_{\mathrm{TH}}-\Delta V_{\mathrm{TH}}\right)}^{2}$$

where Δ*A* is the transconductance mismatch that results in a gain error and Δ*V*_TH_ is the threshold voltage mismatch that results in voltage offsets [15]. The current through the NOD (*I*_data_cm1_), including the mismatch effects, is given by

(7) $$I_{\mathrm{data}\_\mathrm{cm}1}=(A+2\Delta A){\left(V_{\mathrm{GS}}-V_{\mathrm{TH}}-2\Delta V_{\mathrm{TH}}\right)}^{2}$$

The current through the POD is similar to *I*_data_cm0_. Furthermore, PMOS-based circuits are vulnerable to mismatches because of poor gate oxide capacitance matching and high mobility variations [16]. Thus, when numerous current mirrors are used, the current mismatch increases, as expressed in (7). The standard deviations of *V*_data0,1_ (*V*_ref0,1_) shown in Figure 1c,d are relatively large because of the many steps in the current mirror and small slope of the POD0,1 (POR0,1) I-V curve. Consequently, even though the HSSC doubles *μ*_ΔV0,1_ using the DRV technique, it cannot guarantee a sufficient read yield in deep submicron technology.

Figure 1e,f show the SPSC [10] at states 0 and 1, respectively. The SPSC uses a split path to employ the DRV technique and achieves PMOS degeneration to reduce the variation. Figure 2c shows that the SPSC successfully modifies *V*_ref_ according to the MTJ state; the subsequent *V*_ref0_ and *V*_ref1_ values that confirm this are presented in Figure 1e,f. Furthermore, to overcome the process variation issue faced by the HSCC, the SPSC utilizes a split-path technique instead of current mirrors. Instead of employing current mirrors to transmit the NCD saturation current to the NOD as HSCC does, the SPSC uses the split path on the NCD source and applies the same gate-to-source voltage to both the NCD and NOD. As a result, even without a current mirror, the saturation currents of the NCD and NOD are equalized. The standard deviations of *V*_data0,1_ (*V*_ref0,1_) presented in Figure 1e,f is smaller than those of the HSCC because the number of current mirrors is reduced. Therefore, among the existing SCs, SPSC is superior in terms of the *RAPY*_CELL_. However, as shown in Figure 1e,f, the standard deviations of *V*_ref0_ and *V*_data1_ remain large (i.e., 106 mV and 101 mV). This is because the data current (*I*_data_) is split in half by the split-path scheme (*I*_data_ = *I*_data_cm_ + *I*_data_sp_), and the lowered current is sensitive to the increased process variations in deep submicron technology.

### 2.2. Proposed TSSC

**Figure 3:**
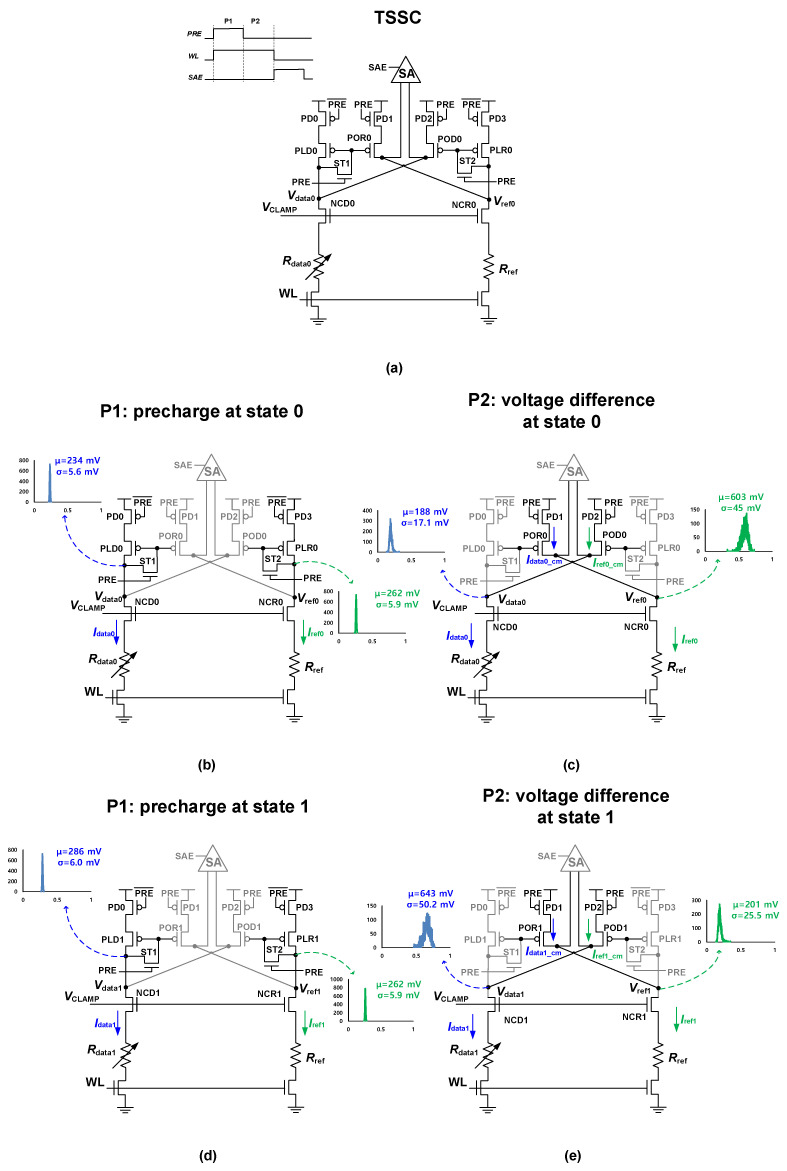
(**a**) Schematic and timing diagram of TSSC. Operations with TSSC voltage distributions in (**b**) phase 1 at state 0, (**c**) phase 2 at state 0, (**d**) phase 1 at state 1, and (**e**) phase 2 at state 1.

**Figure 4:**
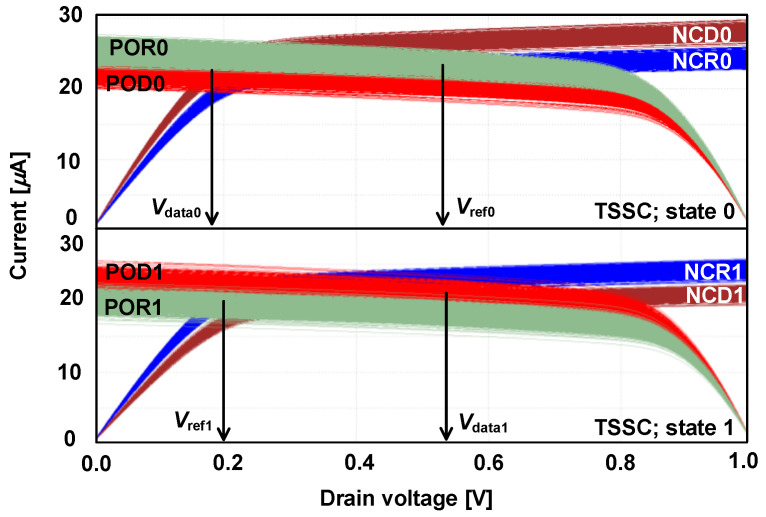
I-V curves of the TSSC with both MOSFET and MTJ process variations for analyzing operating points.

In SPSC, the lowered saturation current caused by the split path is vulnerable against process variations. The TSSC maintains the advantages of SPSC (i.e., the DRV technique, thereby increasing the sensing margin Δ*V*_0,1_) but overcomes the lowered saturation current problem. Figure 3a shows the schematic and timing diagram of the proposed TSSC. The key circuit design differences between the TSSC and SPSC are the inclusion of two transistors as switches (ST1 and ST2) and exclusion of NOD and NOR by implementing a timing-based split-path scheme. To reduce *σ*_ΔV0,1_ by enhancing the current for each path, the MOSFET operating times are controlled by the PRE signal. Figure 3b shows the TSSC operation in phase 1 (P1) at state 0. In P1, the PRE signal is high; thus, PD0, PD3, ST1, and ST2 are turned on at the beginning. The gate voltage of POR0 (POD0) starts to pre-charge as ST1 and ST2 are turned on. PLD0 and PLR0 reach saturation points after a sufficient pre-charge time if *I*_data0_ and *I*_ref0_ are constant in P1, regardless of the process variations in PLD0 (PLR0). At the end of P1, the gate voltage of POR0 (POD0) is kept at the same value as *V*_data0_ (*V*_ref0_). However, POR0 and POD0 cannot operate because PD1 and PD2 are turned off, respectively. Figure 3c shows the TSSC operation in phase 2 (P2) at state 0. At the beginning of P2, the PRE signal is turned off. Thus, PD0, PD3, ST1, and ST2 are turned off, and PD1 and PD2 are turned on. When PLD0 and PLR0 are cut off from *V*_DD_, POR0 and POD0 can operate because the gate voltages of POR0 and POD0 are pre-charged to *V*_data0_ and *V*_ref0_, respectively, in P1. Thus, the saturation current of POR0 is transmitted from NCD0 as the SPSC. However, for TSSC, the saturation current of POR0 is approximately two times larger than that of the SPSC, regardless of process variation. This is because PLD0 is turned off in P2 and there is no current division. In addition, in the TSSC, without current division, the clamp NMOS number is less than that of the SPSC, which is achieved by shifting the split-path position to the drain of the clamp NMOS. Therefore, the TSSC implements the DRV technique with the minimization of *σ*_ΔV0,1_, which is confirmed by the TSSC voltage distribution diagram shown in Figure 3c,e. The process variation in the TSSC can be further decreased compared to the SPSC because in terms of iso-area, the size of MOSFETs in the TSSC can be increased as the number of clamp NMOS is reduced. Figure 3d,e show the TSSC operation at state 1 in P1 and P2, respectively. The TSSC operation at state 1 is nearly identical to that at state 0, with only a change in the MTJ state. Figure 4 shows the TSSC I-V curves, from which *V*_data0,1_ and *V*_ref0,1_ can be estimated. The crossing point in the I-V curves for NCD0 (NCD1) and POD0 (POD1) is the operating point for *V*_data0_ (*V*_data1_). Furthermore, the crossing point in the I-V curves of NCR0 (NCR1) and POR0 (POR1) is the operating point for *V*_ref0_ (*V*_ref1_). *V*_ref_ is successfully modified according to the MTJ state, and operation current is nearly doubled compared to that of SPSC.

## 3. Simulation Results

### 3.1. Simulation Conditions

All simulation results included in this section were obtained using Monte Carlo HSPICE simulations implemented in industry-compatible 28-nm model parameter libraries. The *μ*_SA_OS_ and *σ*_SA_OS_ values for calculating *RAPY*_CELL_ were set to 0 and 20 mV, respectively [17]. Furthermore, a standard deviation of 4% was considered for the MTJ variation. The MTJ model used in this study had an *R*_1_ (*R*_H_, anti-parallel) of 6 kΩ and *R*_0_ (*R*_L_, parallel) of 3 kΩ, considering a tunnel magnetoresistance (TMR) ratio of 100%. For a fair comparison between SCs in terms of iso-area and iso-power, all transistor sizes were chosen such that the layout area (=sum of each transistor’s area (width × length)) of each SC was 1.76 μm^2^. Moreover, the *V*_CLAMP_ for each SC was precisely set such that it generated 20 μA at state 1. In addition, the optimal *R*_ref_ for each circuit was used for architectural analysis.

### 3.2. Results and Comparison

**Figure 5:**
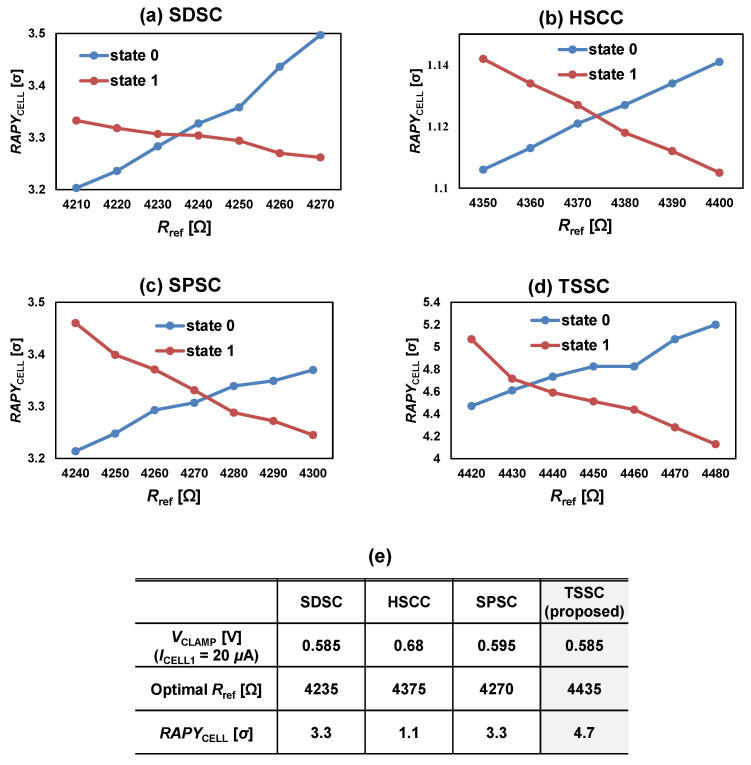
*RAPY*_CELL0_ and *RAPY*_CELL1_ of (**a**) SDSC; (**b**) HSCC; (**c**) SPSC; and (**d**) TSSC with respect to *R*_ref_. (**e**) Optimal *R*_ref_ and *RAPY*_CELL_ of SDSC, HSSC, SPSC, and TSSC.

*RAPY*_CELL0_ and *RAPY*_CELL1_ increased when *R*_ref_ increased and decreased, respectively. Accordingly, the crossing points for *RAPY*_CELL0_ and *RAPY*_CELL1_ were the maximum values of *RAPY*_CELL_. Figure 5 shows *RAPY*_CELL0,1_ for SDSC, HSCC, SPSC, and TSSC with respect to *R*_ref_ when the temperature was set to room temperature (25 °C). The HSCC achieved the lowest *RAPY*_CELL_ value owing to the large output variation due to the current mismatch of the multiple current mirrors. Despite the large *μ*_ΔV0,1_, the SDSC and SPSC exhibited the same value for *RAPY*_CELL_ in terms of iso-area because the SDSC transistor size was twice as large as that of the SPSC. The *RAPY*_CELL_ value of TSSC was the largest compared with that of the conventional SC because the DRV technique was successfully implemented and a timing-based split-path scheme overcame the lowered data current problem faced by SPSC.

**Figure 6:**
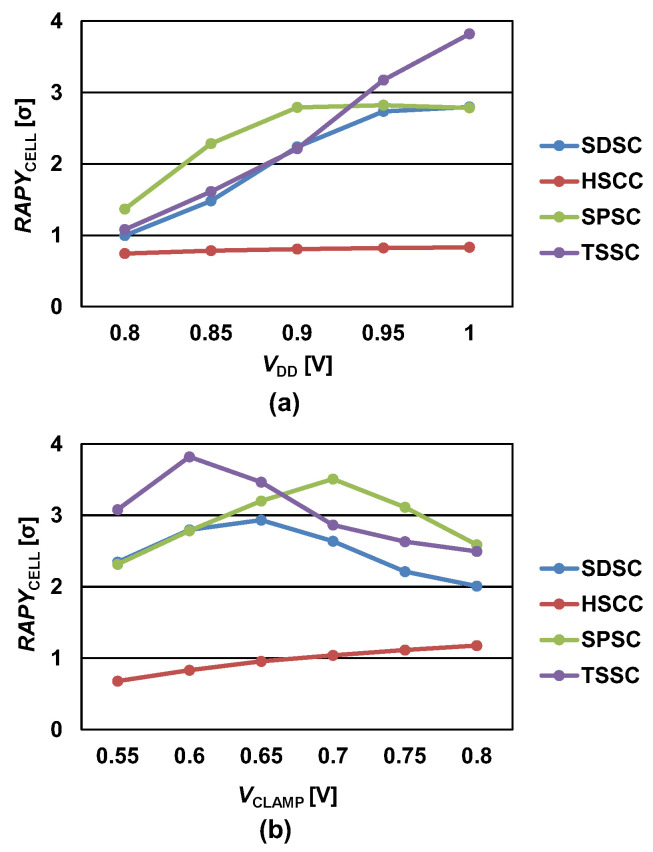
*RAPY*_CELL_ of SDSC, HSCC, SPSC, and TSSC with respect to (**a**) *V*_DD_ and (**b**) *V*_CLAMP_.

Figure 6 shows the *RAPY*_CELL_ for SDSC, HSCC, SPSC, and TSSC with respect to *V*_DD_ and *V*_CLAMP_. To analyze the SCs in terms of iso-power, *V*_CLAMP_ for each SC was precisely set such that it generates 20 μA at state 1. The temperature range was set in the range from −45 to 90 °C, and the worst case was chosen for *RAPY*_CELL_ calculation. As shown in Figure 6a, the HSCC exhibited minimal variations in *RAPY*_CELL_ when *V*_DD_ increased because *σ*_ΔV0,1_ was excessively large owing to the number of current mirrors. When *V*_DD_ was less than 0.9 V, *RAPY*_CELL_ for the SPSC was greater than that for the TSSC. In the TSSC, *RAPY*_CELL_ was significantly reduced at low *V*_DD_ because the decrease rate of *μ*_ΔV0,1_ was high. When *V*_DD_ was greater than 0.9 V, the TSSC achieved a large value for *RAPY*_CELL_ because the increase rate of *μ*_ΔV0,1_ was higher than that of *σ*_ΔV0,1_. Figure 6b shows *RAPY*_CELL_ with respect to *V*_CLAMP_ when *V*_DD_ = 1.0 V. *RAPY*_CELL_ value’s reliance on *V*_CLAMP_ is not linear because when *V*_CLAMP_ is low, the operating current is not enough, and when *V*_CLAMP_ is high *μ*_ΔV0,1_ decreases because of the decreased *r*_O_PLD_. Thus, *V*_CLAMP_ has an optimal value that maximizes the read yield. The HSCC has low *RAPY*_CELL_ and no optimal value according to *V*_CLAMP_ in the range from 0.55 to 0.8 V. The optimal *V*_CLAMP_ values for SDSC and SPSC are 0.65 V and 0.7 V, respectively. However, STT-MRAM requires low sensing current to prevent read disturbances. An unintentional write operation occurs during a read operation when the critical MTJ switching current is lower than the read current [11]. In the TSSC, the optimal *V*_CLAMP_ value, 0.6 V, is lower than that of conventional SCs, and the *RAPY*_CELL_ value is the highest. Therefore, the TSSC is suitable to be implemented in STT-MRAM, which requires low sensing current. 

**Figure 7:**
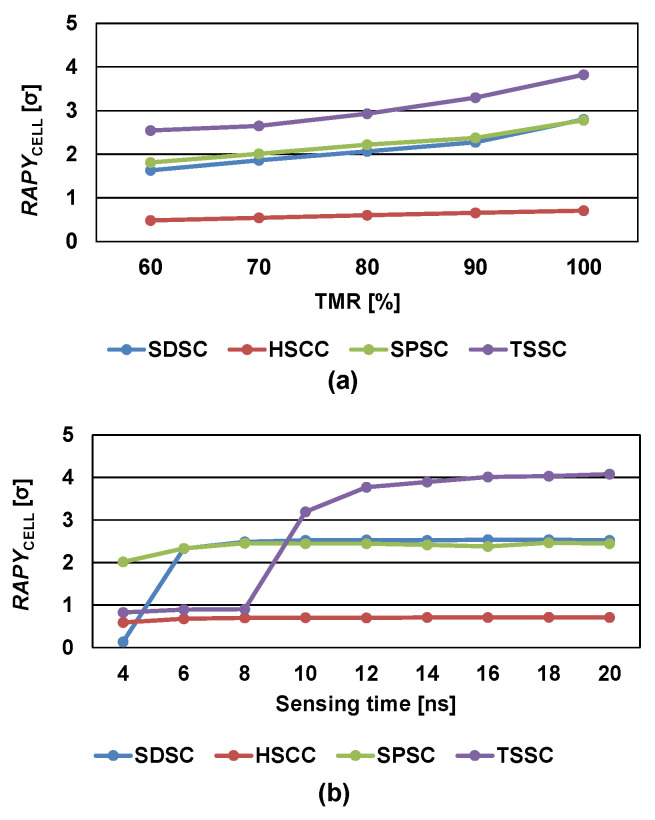
*RAPY*_CELL_ of SDSC, HSCC, SPSC, and TSSC with respect to (**a**) TMR of MTJ and (**b**) sensing time.

Figure 7a illustrates the *RAPY*_CELL_ values of the SDSC, HSCC, SPSC, and proposed TSSC with respect to the TMR of MTJ when *V*_DD_ is set to 1 V and *V*_CLAMP_ is set to 0.6 V. The temperature range was set in the range from −45 to 90 °C and the worst case was chosen for *RAPY*_CELL_ calculation. TMR is calculated as (*R*_1_ − *R*_0_)/*R*_0_ and ideally assumes a high values because it influences the sensing speed, read margin, and noise margin of the memory cell. However, the SCs for STT-MRAM must be built to compensate for process-related fluctuations in the TMR value. As shown in Figure 7a, for low sensing current applications, even when the TMR value is decreased to 60%, the proposed TSSC maintains a greater *RAPY*_CELL_ value compared to existing SCs. Figure 7b plots the *RAPY*_CELL_ as a function of sensing time when *V*_DD_ was set to 1 V and *V*_CLAMP_ was set to 0.6 V. Because the TSSC uses a two-phase sensing operation for the timing-split-based DRV technique, it requires sufficient time for charging and discharging. Thus, a sharp increase in *RAPY*_CELL_ can be observed at approximately 8 ns, and the *RAPY*_CELL_ value is saturated at approximately 14 ns. The existing designs are not sensitive to the sensing time compared to the proposed TSSC, which provides more accurate sensing at the expense of increased sensing times. 

**Figure 8:**
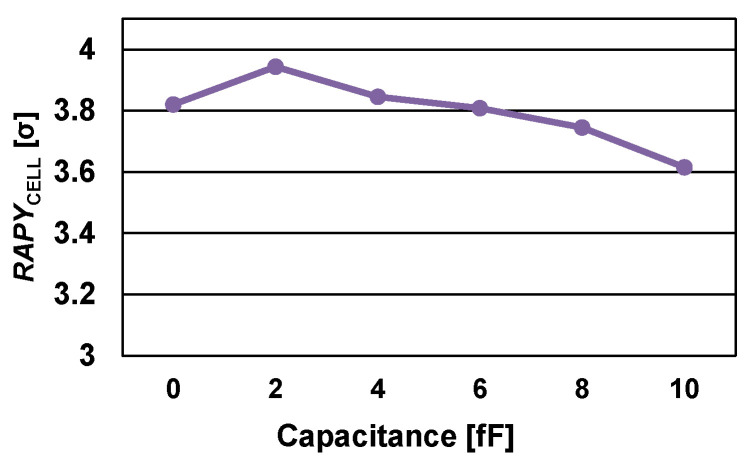
*RAPY*_CELL_ of TSSC with respect to capacitance value when an additional capacitor is assumed.

As mentioned earlier, the TSSC exploits the gate capacitance of the PLD, POR, POD, and PLR transistors to accumulate charge during P1, which raises the question of whether the capacitor-less design is better than using a capacitor. Figure 8 shows the TSSC *RAPY*_CELL_ value with respect to the additional capacitor capacitance when two capacitors are added at the gates of the PLD and PLR. The *RAPY*_CELL_ value increases until the additional capacitor capacitance reaches 2 fF but the increment is insignificant and starts to decrease as the capacitance increases further because of the limited sensing time. Moreover, if a capacitor is added to the design, the sizes of all transistors need to be reduced in terms of iso-area, which results in a decrease in the gate capacitance and performance degradation. Accordingly, Figure 8 indicates that the gate capacitances of PLD, POR and POD, PLR pairs are sufficient for the TSSC to accumulate charge during P1 to mirror the data current during P2. 

Table 1 summarizes the simulation results and compares the proposed TSSC with conventional SCs. The simulation to calculate the average power consumption (*P*_AVG_) used and the *V*_CLAMP_ and *R*_ref_ values are presented in Figure 5. In both states 0 and 1, the TSSC achieved the lowest *P*_AVG_ compared with the conventional SCs. However, the proposed TSSC requires a longer sensing time because of its charging and discharging times. As shown in Table 1, the TSSC can achieve a 42% higher *RAPY*_CELL_ value compared to that of SPSC. Because of the read disturbance, sensing circuitries are required to work with low sensing currents. Moreover, the *RAPY*_CELL_ value of the proposed TSSC is the highest in low-current sensing tasks. Therefore, the TSSC is suitable for STT-MRAM applications, which require low sensing currents.

## 4. Conclusions

In this study, we proposed a TSSC using the DRV technique and novel split path over time, which maintains a large *μ*_ΔV0,1_ and reduces *σ*_ΔV0,1_. The proposed TSSC can reduce the *V*_TH_ variation effects by increasing the current and reducing the transistor mismatch. The simulation results indicate that conventional SCs exhibit low read yield because of small *μ*_ΔV0,1_ or large *σ*_ΔV0,1_ values. In contrast, the TSSC obtains the greatest read yield in the 28-nm process technology.

## Figures and Tables

**Table 1:** Summary of the simulation results in terms of iso-area (identical Tr. size) and iso-power.

	SDSC [11]	HSSC [13]	SPSC [10]	TSSC (Proposed)
Process technology	28 nm			
DRV technique	X	O	O	O
*I*_CELL_ [μA] @ state 1	20.0			
Layout Area of SC [μm^2^] @ iso-area (Layout Area of SC [μm^2^] @ identical Tr. size^(1)^)	1.76 (1.28)	1.76 (3.04)	1.76 (2.56)	1.76 (1.76)
*RAPY*_CELL_^(2)^ [*σ*] @ *V*_DD_ = 1.0 V, iso-area (*RAPY*_CELL_^(2)^ [*σ*] @ *V*_DD_ = 1.0 V, identical Tr. size)	3.3 (3.12)	1.1 (1.19)	3.3 (3.36)	4.7 (4.7)
*RAPY*_CELL_^(3)^ [σ] @ V_DD_ = 1.0 V, iso-area (*RAPY*_CELL_^(3)^ [σ] @ V_DD_ = 1.0 V, identical Tr. size)	2.80 (2.68)	0.83 (0.89)	2.78 (2.79)	3.82 (3.82)
Average sensing time [ns] @ V_DD_ = 1.0 V, iso-area (Average sensing time [ns] @ V_DD_ = 1.0 V, identical Tr. size)	7 (7)	8 (8)	8 (8)	14 (14)
*P*_AVG_ [μA] @ state 0, V_DD_ = 1.0 V, iso-area (*P*_AVG_ [μA] @ state 0, V_DD_ = 1.0 V, identical Tr. size)	50.1 (48.1)	153 (213)	53.0 (62.9)	48.9 (48.9)
*P*_AVG_ [μA] @ state 1, V_DD_ = 1.0 V, iso-area (*P*_AVG_ [μA] @ state 1, V_DD_ = 1.0 V, identical Tr. size)	46.6 (44.7)	137 (188.7)	47.0 (55.8)	44.0 (44.0)

(1) For the identical transistor size (width/length), load PMOS of 2 μm/0.1 μm, clamp NMOS of 4 μm/0.1 μm, and switch of 0.3 μm/0.05 μm were used. (2) Temperature was set to room temperature (25 °C) and *V*_CLAMP_ was set to the values depicted in Figure 5. (3) The worst *RAPY*_CELL_ value was calculated by comparing the results obtained at 45 °C and 90 °C when *V*_CLAMP_ was set to 0.6 V.

## Data Availability

Data is contained within the article.
